# Comparison of intima-media thickness and ophthalmic artery resistance index for assessing subclinical atherosclerosis in HIV-1-infected patients

**DOI:** 10.1186/1476-7120-9-9

**Published:** 2011-04-01

**Authors:** Pierfrancesco Grima, Marcello Guido, Roberto Chiavaroli, Antonella De Donno, Mariangela Tana, Antonella Zizza

**Affiliations:** 1 Division of Infectious Diseases, HIV Centre, S. Caterina Novella Hospital, via Roma, 73013 Galatina, Italy; 2 Laboratory of Hygiene, Department of Biological and Environmental Sciences and Technologies, Faculty of Sciences, University of Salento, Lecce, Italy; 3 National Research Council, Institute of Clinical Physiology, Lecce, Italy

**Keywords:** HIV, cardiovascular risk, atherosclerosis, intima-media thickness, ophthalmic artery resistance index

## Abstract

**Background:**

Human immunodeficiency virus (HIV) infection and antiretroviral treatment are associated with metabolic and cardiovascular complications that potentially increase the risk of atherosclerosis and cardiovascular disease in this population. Measurement of arterial wall thickness has been used as a surrogate of extent, severity and progression of atherosclerosis. A cross-sectional cohort study was performed to compare the validity of two non-invasive arterial measures: carotid intima-media thickness (IMT), a parameter of atherosclerosis, and ophthalmic artery resistance index (OARI), an index of occlusive carotid artery disease.

**Methods:**

A total of 95 patients receiving highly active antiretroviral therapy (HAART) for more than 12 months were consecutively enrolled. IMT and OARI were measured by 7.5 MHz linear probe.

**Results:**

There was a significant linear increase in IMT and OARI values as the grade of cardiovascular risk (0.70 and 0.69 for very low risk, 0.86 and 0.72 for low risk and 0.98 and 0.74 for medium/high risk, p < 0.001). A IMT > 0.83 and an OARI > 0.72 were the most discriminatory values for predicting a cardiovascular risk ≥ 10% (sensibility 89.6% and 75.8%; sensitivity 70.5% and 68.4%; p < 0.001).

**Conclusions:**

Our data indicate that OARI may have a potential as a new precocious marker of subclinical atherosclerosis in HIV-1-infected patients.

## Background

The introduction of highly active antiretroviral therapy (HAART) has dramatically changed the prognosis of human immunodeficiency virus (HIV) infection [1]. In this scenario, cardiovascular diseases have gained importance as possible causes of morbidity and mortality [2,3]. Cardiovascular disease is often correlated to the development of atherosclerosis with plaque formation on arterial walls [4].

Measurement of arterial wall thickness has been used as a surrogate of extent, severity and progression of atherosclerosis[5]. Carotid intima-media thickness (IMT) is a measure of anatomic disease and a significant predictor of acute coronary events [5,6] associated with cardiovascular risk factors [5,7,8].

Moreover, carotid IMT is associated with cardiovascular disease (CVD) risk factors, prevalent CVD, incident CVD and the degree of atherosclerosis [9-12]. These observations support the concept that carotid IMT measurement could be used as a surrogate marker of atherosclerosis.

The ophthalmic artery (OA) is the first major branch of the internal carotid artery, so changes in OA blood flow have provided insight into various vascular disorders, including carotid artery stenosis [13]. OA Doppler has anatomical advantages due to the absence of ultrasonic obstacles associated with systemic atherosclerosis, with previous data showing correlation between alterations of the OA and systemic cardiovascular disease [14,15].

There are limited data on the relationship between carotid IMT or OA blood flow and cardiovascular risk factors in HIV patients [16].

Therefore, the aim of this study is to investigate whether OA Doppler imaging could have clinical potential for evaluating the severity of cardiovascular risk in HIV-1+ infected patients, with a better reproducibility than IMT assessment.

## Methods

This study was approved by the local institutional Ethics Committee and all patients approached for the study gave written consent to participate.

HIV-1-infected patients referred to our outpatient center between 1 January and 31 June 2009 were considered for this study. Inclusion criteria were: age 18-70 years, proven HIV-1 infection, and receiving HAART for more than 12 months. Exclusion criteria were: active AIDS defining illness, diabetes mellitus, active drug abuse, alcohol abuse (defined as alcohol consumption of more than 30 gr/day). Patients requiring systemic chemotherapy, radiation therapy or systemic steroids were excluded). An in-depth assessment was performed, including HIV disease history, other co-morbid conditions, medication exposure and measurement of blood pressure (determined using a sphygmomanometer with the subjects at rest in a sitting position). Smokers were defined as individuals smoking more than 5 cigarettes/day at least during the past year (in our cohort all the patients who smoked declared they smoked > 5 cigarettes/day). CD4+ cell counts, HIV RNA load, total serum cholesterol level, high-density lipoprotein (HDL) cholesterol level, glucose and triglyceride levels were evaluated at baseline after a 12-h overnight fast. In all patients the 10-year risk of having a heart attack was estimated by the Framingham equation (available at the following on-line address: http://hp2010.nhlbihin.net/atpiii/calculator.asp). Patients were classified as very low, low, or medium/high risk if Framingham risk scores were < 5%, 5-9% and ≥ 10% respectively [17].

**Figure 1:**
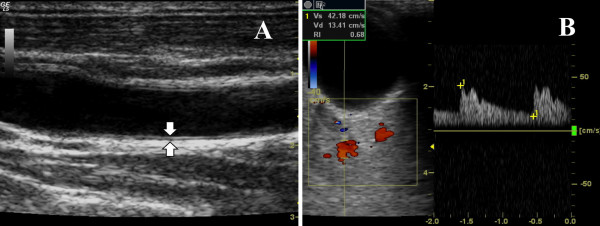
**IMT and OARI measurement**. (A) Common carotid intima-media thickness. Longitudinal scan obtained at 1 cm proximal to the origin of the carotid bulb shows the IMT (arrows). (B) Ophtalmic artery resistence index. Both measurements were evaluated by ultrasound frequency of 7.5 mHz.

For ultrasound measurement of IMT and OARI a Logiq 5 ultrasound scanner (General Electric Medical Systems, Wallingford, Connecticut, USA) was used. Sonographic evaluations were performed by a single trained sonographer blinded to the patients' data. The patients lay supine in a quiet, dark room. The right and left common carotid arteries were examined with the head in the midline position tilted slightly upward. A 7.5 MHz linear probe was used. The probe was placed so that the near and far walls were parallel to it and lumen diameter was maximized in the longitudinal plane. The IMT was defined as the distance between the media-adventitia interface and the lumen-intima interface and was measured at about 1 cm proximal to the bifurcation of the common carotid artery. Two parallel echogenic lines separated by an anechoic space can be visualized in the anterior wall of the carotid (Figure 1A).

OARI was measured with the patient lying supine with eyes closed, using an ultrasound frequency of 7.5 MHz and by averaging the readings from at least three consecutive waveforms. The transducer was applied to the closed upper eyelid using a thick layer of acoustic gel, minimizing the pressure on the globe (Figure 1B).

The reproducibility of IMT and OARI measurement was evaluated by triple determinations in 20 subjects other than the enrolled patients.

Continuous variables were reported as the mean ± standard deviation (SD) and categorical factors were reported as percentages. One-way analysis of variance (ANOVA) models were used to examine differences in carotid IMT or OARI by Framingham risk group classification. Multiple regression analysis was used to assess the independent association between carotid IMT or OARI and traditional Framingham cardiovascular risk factors of age, male gender, current smoking status, systolic blood pressure, total and HDL cholesterol. The most discriminant cutoffs were calculated by receiver operating characteristic (ROC) curves.

We evaluated the relationship between carotid IMT and OARI using the Pearson correlation co-efficient. Statistical calculations were performed with MedCalc software, version 11.4.1.0. A p-value < 0.05 was considered to be statistically significant.

## Results

**Figure 2:**
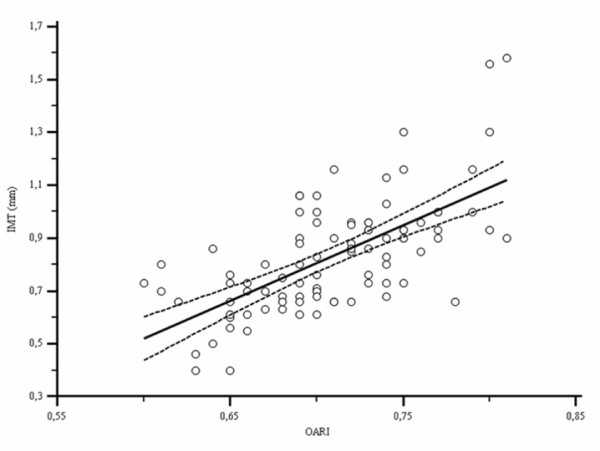
**Linear regression curve of relation between IMT and OARI**. Dotted line, 95% confidence interval; IMT, intima-media thickness; OARI, ophthalmic artery resistance index

Ninety-five patients (74 men and 21 women; mean age 45.2 ± 8.6 years; range 23-70 years) were recruited between January 1 and June 31, 2009. Demographic, epidemiological, clinical, metabolic and ultrasonographic characteristics of patients are shown in Table 1. Table 2 shows different antiretroviral drugs used by our study population. There were 75 (78.9%) patients treated with Tenofovir, 12 (12.6%) with Abacavir and Lamivudine, and 76 (80%) with Emtricitabine. Non-nucleoside reverse transcriptase inhibitor (NNRTIs) therapy included Efavirenz in 36 (37.8%) patients and Nevirapine in 12 (12.6%) patients. Protease inhibitors (PI) included Lopinavir/Ritonavir in 5 (5.2%) patients, Darunavir/Ritonavir in 16 (16.8%), Atazanavir/Ritonavir in 13 (13.6%) and Atazanavir without Ritonavir in 4 (4.2%). There were 2 (2.1%) patients treated with Maraviroc and 10 (10.5%) with Raltegravir. We found a significant positive correlation between IMT and OARI (r = 0.66, p < 0.0001; Figure 2).

**Table 1:** Demographic, clinical, metabolic and ultrasonographic characteristics of HIV-1-infected patients

Variable	Estimate
Age (years)	45.2 ± 8.6
Sex (M/F)	74/21
Current smoker	50 (52.6%)
Systolic pressure (mmHg)	121 ± 15
**HIV exposure**	
Homosexual	32 (33.6%)
Heterosexual	34 (35.7%)
IDU	28 (29.47%)
HCV co-infection	25 (26%)
CD4 count (cells/ml)	525.7 ± 249.7
CD4 percentage (%)	27.5 ± 10.7
CD4 nadir count (cells/ml)	208.8 ± 120
CD4 nadir percentage (%)	17.6 ± 7.9
HIVRNA load (log10 copies)	1.5 ± 0.8
Duration of HIV infection (years)	10.3 ± 7.8
Duration of HAART (months)	76.8 ± 46.2
Total cholesterol (mg/dl)	179.9 ± 46.1
HDL cholesterol (mg/dl)	45.8 ± 12.9
Glucose (mg/dl)	97.3 (21.2)
Right common carotid IMT (mm)	0.83 ± 0.21
Left common carotid IMT (mm)	0.85 ± 0.21
Right OARI	0.70 ± 0.04
Left OARI	0.71 ± 0.04

IDU, intravenous drug users; HAART, highly active antiretroviral therapy; HDL, high density lipoprotein; IMT, intima-media thickness; OARI, ophthalmic artery resistance index. All data are expressed as mean ± standard deviation. A p-value of < 0.05 was considered to be statistically significant.

**Table 2:** Antiretroviral treatment used in ninety-five HIV-1-infected patients

Antiretroviral drugs	Number of patients (%)
TDF	75 (78.9)
ABC	12 (12.6)
3TC	12 (12.6)
FTC	76 (80)
NVP	12 (12.6)
EFV	36 (37.8)
LPV/RTV	5 (5.2)
ATV/RTV	13 (13.6)
ATV	4 (4.2)
DRV/RTV	16 (16.8)
RAL	10 (10.5)
MVC	2 (10.5)

TDF, tenofovir; ABC, abacavir; 3TC, lamivudine; FTC, emtricitabine; NVP, nevirapine; EFV, efavirenz; LPV/RTV, lopinavir/ritonavir; ATV/RTV, atazanvir/ritonavir; DRV/RTV, darunavir/ritonavir; RAL, raltegravir; MVC, maraviroc. All data are expressed as number (%) of patients.

**Figure 3:**
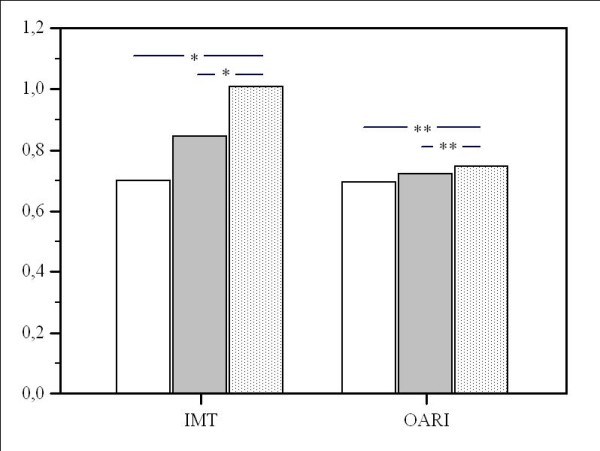
**Characteristics for data by Framingham risk group**. White bar, very low cardiovascular risk (< 5%); grey bar, low cardiovascular risk (5-9%); dotted bar, medium/high cardiovascular risk (≥ 10%); black lines, Standard deviation; IMT, intima-media thickness; OARI, ophthalmic artery resistance index. * p < 0.0001; **p < 0.01

We obtained a good reproducibility of IMT (ICC = 0.90; 95% CI 0.81 -0.95 for single measures and ICC = 0.96; 95% CI 0.92 -0.98 for average measures) and OARI (ICC = 0.94; 95% CI 0.89-0.97 for single measures and ICC = 0.98; 95% CI 0.86-0.99 for average measures). Stratifying by Framingham risk group, there was a significant linear increase of carotid IMT or OARI as the risk group classification (Table 3). Carotid IMT and OARI differed significantly between risk groups from ANOVA analysis (p < 0.001) (Figure 3). Using carotid IMT or OARI as the dependent variable in regression analysis, age (r:0.57, p < 0.0001 and r:0.57, p < 0.0001 respectively), current smoking status (r:0.44, p < 0.01 and r:0.30, p < 0.05 respectively) and systolic pressure (r:0.48, p < 0.01 and r:0.36, p < 0.05 respectively) were independent factors associated with IMT and OARI. Neither total cholesterol nor HDL cholesterol were statistically significantly associated with IMT or OARI.

**Table 3:** Characteristics for data by Framingham risk group

Characteristics	A (n = 37)	B (n = 23)	C (n = 35)	p-value
IMT (mm)	0.70 ± 0.2	0.86 ± 0.21	0.98 ± 0.2	< 0.0001*
OARI (mm)	0.69 ± 0.05	0.72 ± 0.04	0.74 ± 0.04	< 0.001**

A, very low cardiovascular risk (< 5%); B, low cardiovascular risk (5%-9%); C, medium/high cardiovascular risk (≥ 10%). IMT, intima-media thickness; OARI, ophthalmic artery resistance index. Data are expressed as mean ± standard deviation. *A vs B and B vs C; ** A vs B. A p-value of < 0.05 was considered to be statistically significant.

ROC curves indicated that the most discriminant IMT or OARI value for predicting a medium/high cardiovascular risk was > 0.83 mm (sensitivity 89.6%, specificity 70.5%, AUC 0.84, Standard error 0.04, p < 0.0001) and > 0.72 (sensitivity 75.8%, specificity 68.3%, AUC 0.73, Standard error 0.06, p < 0.001) respectively. There was no statistically significant difference between IMT and OARI ROC curves (difference 0.11, standard error 0.07; p = 0.12)

## Discussion

Several reports have shown that HIV-1-infected patients have an increased risk of cardiovascular disease [18,19]. Ultrasound may be an important step towards selecting patients at medium/high risk for developing cardiovascular disease, allowing early intervention, and thus minimizing the impact of clinical complications resulting from this condition. Measurement of carotid IMT by ultrasonography is a well established method for assessing sub-clinical atherosclerosis in the HIV-negative population and it is the only non-invasive imaging recommended by the American Heart Association for inclusion in risk assessment for cardiovascular disease [20]. The manual measurement of IMT is the most common technique used in clinical practice, even though it is time-consuming and the results from these readings may be biased by the lack of expertise or by some subjective judgment of the observer. Thus, automated measurement procedures have been developed [21,22]. The development of automated methods for measuring IMT in standard ultrasound equipment has contributed importantly to better reproducibility of results between different observers as well as reducing considerably the time needed for image evaluation. However, the mean difference between the automated and manual methods for IMT measurement was not clinically relevant in any of the carotid segments evaluated in the majority of cases, and carries no systematic errors [23].

On the other hand, the automated method also enables us to reliably compare online data with other previously published reports for different populations in percentiles, as recently proposed by the American Society of Echocardiography [24].

IMT values correlate well with pathological measurements and are potent predictors of myocardial infarction and stroke. For each 0.1 mm increase in common carotid IMT the risk of acute myocardial infarction increases by 11% [9].

IMT is a feature of arterial wall aging that is not synonymous with subclinical atherosclerosis, but is related to it because the cellular and molecular alterations that underlie intima-media thickening have been implicated in the development, progression, or both of atherosclerosis [24].

The ophthalmic artery is the first major branch of the internal carotid artery, and changes in blood flow have provided new insights into various vascular disorders including carotid artery stenosis and metabolic disorders [17,25]. Orbital circulation changes with varying degrees of carotid stenosis [26] were observed, with a significant relation between orbital velocity changes and carotid occlusive disease [27]. Studies on carotid artery stenosis have shown decreased blood flow velocity in the ophthalmic artery when the stenosis was greater than 70% [28], showing that measurement of orbital vessel velocity may be essential for evaluating the distal consequences of carotid artery stenosis [13].

Although carotid artery ultrasound evaluation is accurate for diagnosis of carotid stenosis, additional images may provide further information on the hemodynamics of the internal carotid artery, including the velocities and pulsatility indices of the orbital arteries, which may reflect the hemodynamics of proximal carotid stenosis, the flow direction of the ophthalmic artery and the possibility of a hemodynamically significant lesion distal to the bifurcation of the carotid artery [25].

Delineating the direction of flow in the ophthalmic artery is important in the assessment of patients with cerebrovascular disease and may enhance our understanding of the pathogenesis of stroke [25].

OARI offers important advantages due to the absence of ultrasound obstacles and the vertical angle, which differs from the parallel-signaling of the carotid artery [29].

Furthermore, it may provide information on the hemodynamics of the internal carotid artery and orbital arteries [25] and it has clinical potential for the evaluation of the severity of coronary artery disease (CAD). High levels of OARI seem to reflect diminished arterial compliance caused by systemic atherosclerosis [29].

We used the manual method of IMT assessment according to the most of the studies on IMT evaluation in HIV-1 infected population, to reliably compare data with other published reports. Moreover, as previously demonstrated, we had low intra-observer variability for IMT ultrasonographic measurements [16,30]

Our results regarding the relationship between OARI and atherosclerosis are similar to data of a study [31] that showed the association of ophthalmic artery Doppler flow with systemic arterial compliance. It was shown that a high grade of coronary atherosclerosis is associated with decreased distensibility and loss of elastic inhomogeneity of arteries, resulting in increased pulse wave velocity [32].

In this study we found that OARI could be a clinically significant indicator of medium/high cardiovascular risk in HIV-1 infected patients with low intra-operator variability, reflecting vascular resistance resulting from atherosclerotic functional changes.

OARI evaluations may be an important step towards selecting patients with significant risk for developing cardiovascular disease, allowing early intervention.

## Conclusion

To our knowledge, this is the first study to compare OARI and IMT in order to show that OARI could be considered a surrogate of cardiovascular risk in HIV-1-infected patients.

OARI provides further hemodynamic information and it could be used to complement ultrasonographic assessment of atherosclerosis. Examination of the orbital arteries with color-coded Doppler imaging is a simple, rapid, reproducible and non-invasive method.

Moreover, ophthalmic artery Doppler imaging has a potential role in assessing the severity of cardiovascular diseases.

As this study used a cross-sectional design, prospective designed studies might demonstrate further clinical value of OARI, such as the predictive diagnosis of coronary artery disease in patients with multiple risk factors.

## Abbreviations

IMT: carotid intima-media thickness; OARI: ophthalmic artery resistance index; HAART: highly active antiretroviral therapy; HIV: human immunodeficiency virus; OA: ophthalmic artery; HDL: high-density lipoprotein; SD: standard deviation; ANOVA: analysis of variance, ROC: receiver operating characteristic; NNRTIs: Non-nucleoside reverse transcriptase inhibitors; PI: Protease inhibitors

## Competing interests

The authors declare that they have no competing interests.

## Authors' contributions

PG wrote the manuscript; RC and MT performed the ultrasound examinations; AZ, MG and ADD participated in writing the manuscript and performed the statistical analysis. All authors critically revised the manuscript. All authors read and approved the final manuscript.
